# Human cerebrovascular contractile receptors are upregulated via a B-Raf/MEK/ERK-sensitive signaling pathway

**DOI:** 10.1186/1471-2202-12-5

**Published:** 2011-01-11

**Authors:** Hilda Ahnstedt, Hans Säveland, Ola Nilsson, Lars Edvinsson

**Affiliations:** 1 Division of Experimental Vascular Research, Department of Clinical Sciences, Lund University, Lund, Sweden; 2 Department of Neurosurgery, Lund University Hospital, Lund, Sweden

## Abstract

**Background:**

Cerebral ischemia results in a rapid increase in contractile cerebrovascular receptors, such as the 5-hydroxytryptamine type 1B (5-HT_1B_), angiotensin II type 1 (AT_1_), and endothelin type B (ET_B_) receptors, in the vessel walls within the ischemic region, which further impairs local blood flow and aggravates tissue damage. This receptor upregulation occurs via activation of the mitogen-activated protein kinase pathway. We therefore hypothesized an important role for B-Raf, the first signaling molecule in the pathway. To test our hypothesis, human cerebral arteries were incubated at 37°C for 48 h in the absence or presence of a B-Raf inhibitor: SB-386023 or SB-590885. Contractile properties were evaluated in a myograph and protein expression of the individual receptors and activated phosphorylated B-Raf (p-B-Raf) was evaluated immunohistochemically.

**Results:**

5-HT_1B_, AT_1_, and ET_B _receptor-mediated contractions were significantly reduced by application of SB-590885, and to a smaller extent by SB-386023. A marked reduction in AT_1 _receptor immunoreactivity was observed after treatment with SB-590885. Treatment with SB-590885 and SB-386023 diminished the culture-induced increase of p-B-Raf immunoreactivity.

**Conclusions:**

B-Raf signaling has a key function in the altered expression of vascular contractile receptors observed after organ culture. Therefore, specific targeting of B-Raf might be a novel approach to reduce tissue damage after cerebral ischemia by preventing the previously observed upregulation of contractile receptors in smooth muscle cells.

## Background

Although cerebral ischemia is a leading cause of morbidity and mortality worldwide, few therapeutic advances appear to be of value in the clinic [1]. We have observed an inherent tendency of the vasculature to undergo phenotypic changes as a response to cerebral ischemia [2]. Therefore, the cerebral vessels show transcriptional upregulation of the vasoconstrictive G-protein coupled receptors (GPCRs) 5-hydroxytryptamine type 1B (5-HT_1B_), angiotensin II type 1 (AT_1_) and endothelin type B (ET_B_) after experimental subarachnoid hemorrhage (SAH) or after focal ischemic stroke [3,4]. Identical receptor upregulation has been observed in patients that died of stroke [5]. In both types of experimental stroke, the receptor upregulation is mediated via the mitogen-activated protein kinase pathway MEK/ERK1/2 [6,7]. A similar type of receptor upregulation can be achieved experimentally by incubating isolated arteries in serum-free culture medium at 37°C for 12 to 48 h [8].

The first signaling molecule in the MEK/ERK1/2 pathway, Raf, is a serine/threonine kinase existing in three different isoforms (A-, B-, and C-Raf) with a common activator, Ras, and a single known common substrate, MEK. Even though MEK is the common substrate, experiments on Raf knock-out mice show isoform-specific functions for A-, B-, and C-Raf [9]. B-Raf is the only isoform that is strongly activated by Ras alone [10] and the most active isoform when it comes to phosphorylating MEK *in vitro *[11]. We therefore designed this study to examine the role of the B-Raf isoform in inducing the observed GPCR alterations seen after cerebral ischemia. Two previously characterized B-Raf selective inhibitors were used in this study, SB-386023 [12] and SB-590885 [13]. The inhibitors are both small ATP competitive inhibitors with high selectivity for B-Raf when tested against a panel of related protein kinases, but are different in that SB-590885 has a higher affinity for B-Raf. We show that culturing human cerebral arteries in the presence of B-Raf inhibitors strongly attenuates 5-HT_1B_, AT_1_, and ET_B _receptor-mediated contractions compared with arteries cultured with vehicle alone. The receptor proteins were evaluated with immunofluorescence and a marked reduction in AT_1 _receptor immunofluorescence was observed after treatment with SB-590885. Additionally, the observed increase in phosphorylated B-Raf (p-B-Raf) immunoreactivity after incubation was diminished after treatment with the B-Raf inhibitors.

## Results

### In vitro pharmacology

Initially, the vessel segments were normalized and stretched to 90% of the internal circumference that a fully relaxed vessel under a transmural pressure of 100 mm Hg would have. The mean normalized internal circumference and standard deviation was 725 ± 297 μm. K^+^-induced contractions did not differ significantly among the three groups; vehicle, SB-386023, and SB-590885 data confirmed that all groups responded similarly to K^+^, excluding the possibility that the B-Raf inhibitors had an effect on the viability of the vessels. E_max _and pEC_50 _values for each group are presented in Table 1.

**Table 1:** Contractile responses to 5-CT, Ang II, and ET-1

			Sigmoidal curve		Biphasic curve			
			
	n	K^+ ^(mN)	E_max _(%)	pEC_50_	E_max(1) _(%)	E_max(2) _(%)	pEC_50(1)_	pEC_50(2)_
*5-CT*								
Vehicle	5	6.87 ± 1.07	39.20 ± 12.09	6.92 ± 0.40				
SB-386023	6	7.42 ± 1.10	25.13 ± 4.75	7.28 ± 0.31				
SB-590885	6	5.45 ± 1.16	11.75 ± 3.43*	6.65 ± 0.25				
*Ang II*								
Vehicle	6	7.16 ± 0.92	46.43 ± 6.78	10.11 ± 0.25				
SB-386023	7	7.08 ± 0.95	26.20 ± 4.37	10.15 ± 0.23				
SB-590885	7	5.88 ± 1.12	11.56 ± 2.72***	9.45 ± 0.96				
*ET-1*								
Vehicle	6	7.16 ± 0.92			36.71 ± 12.09	128.40 ± 6.91	11.74 ± 0.20	9.17 ± 0.18
SB-386023	7	7.08 ± 0.95			25.60 ± 7.40	132.20 ± 8.46	11.73 ± 0.16	8.96 ± 0.20
SB-590885	7	5.88 ± 1.12			7.44 ± 2.44*	147.4 ± 11.04	11.37 ± 0.20	9.07 ± 0.08

Responses were characterized by E_max _values, expressed as percent of 63.5 mM K^+^-induced contraction, and pEC_50 _values. Values are represented as mean ± s.e.m., with *n *representing the number of patients. Statistical analyses were performed using the non-parametric Kruskal-Wallis test and Dunn´s multiple comparison test. *P < 0.05, ***P < 0.001 compared with vehicle. 5-CT - 5-carboxamidotryptamine, Ang II - Angiotensin II, ET-1 - Endothelin-1.

### Contractile responses to 5-carboxamidotryptamine

**Figure 1:**
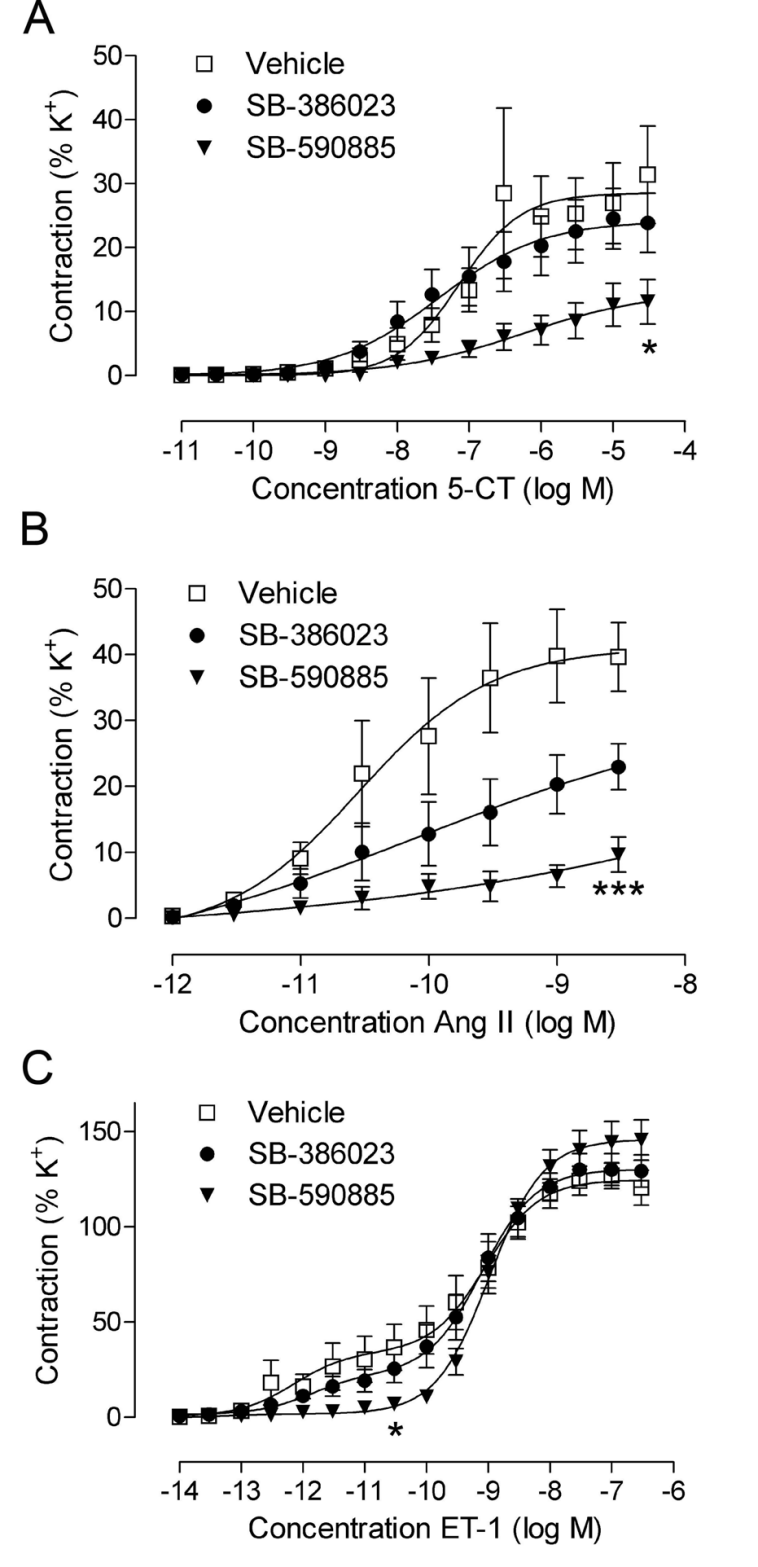
**Contractile responses to 5-carboxamidotryptamine (5-CT) (A), angiotensin II (Ang II) (B), and endothelin-1 (ET-1) (C) in human cerebral arteries**. Effect of organ culture in the presence of vehicle (n = 5 to 6) and in the presence of SB-590885 (n = 6 to 7) and SB-386023 (n = 6 to 7) are illustrated. The receptor-mediated contractions are clearly reduced by SB-590885. Data are expressed as mean ± s.e.m. ET-1 biphasic concentration-response curve: high-affinity phase refers to the endothelin type B (ET_B_) receptor-mediated contraction; low-affinity phase refers to the endothelin type A (ET_A_) receptor-mediated contraction. Statistical analyses are shown for the E_max _values where *P < 0.05 and ***P < 0.001 compared with vehicle. E_max _values and pEC_50 _values for respective concentration-response curves are presented in Table 1.

5-HT_1B _receptor-mediated contraction was studied using cumulative application of 5-carboxamidotryptamine (5-CT). Vessel segments treated with SB-386023 or SB-590885 both showed attenuated contractile responses to 5-CT and gave rise to reduced E_max _values compared with vehicle-treated vessels (Figure 1A). The inhibitory effect was significant for vessels treated with SB-590885, E_max _11.75 ± 3.43% compared with 39.20 ± 12.09% for the vehicle group (P < 0.05, Table 1).

### Contractile responses to angiotensin II

Application of angiotensin II (Ang II) induced a concentration-dependent contractile response at lower concentrations (10^-12 ^- 3·10^-9 ^M) and dilatation at higher concentrations (10^-8 ^- 3·10^-6 ^M, data not shown). The maximum contraction was attenuated after treatment with SB-590885 (11.56 ± 2.72%, P < 0.001) and SB-386023 (26.20 ± 4.37%, not significant) compared with 46.43 ± 6.78% for the control vessels treated with vehicle (Figure 1B, Table 1).

### Contractile responses to endothelin-1

Endothelin-1 (ET-1) gave rise to a biphasic concentration-dependent response indicating the presence of both ET_A _(low affinity) and ET_B _(high affinity) receptors in a manner previously characterized in detail [14]. The high-affinity phase corresponding to ET_B _receptor-mediated contraction was significantly decreased in the presence of SB-590885 (7.44 ± 2.44%, P < 0.05) while SB-386023 did not have a significant effect (25.60 ± 7.40%) compared with vehicle 36.71 ± 12.09% (Figure 1C, Table 1). ET_A _receptor-mediated contractions (low-affinity phase) were not significantly altered by the application of either inhibitor (Figure 1C).

### Immunohistochemistry

**Figure 2:**
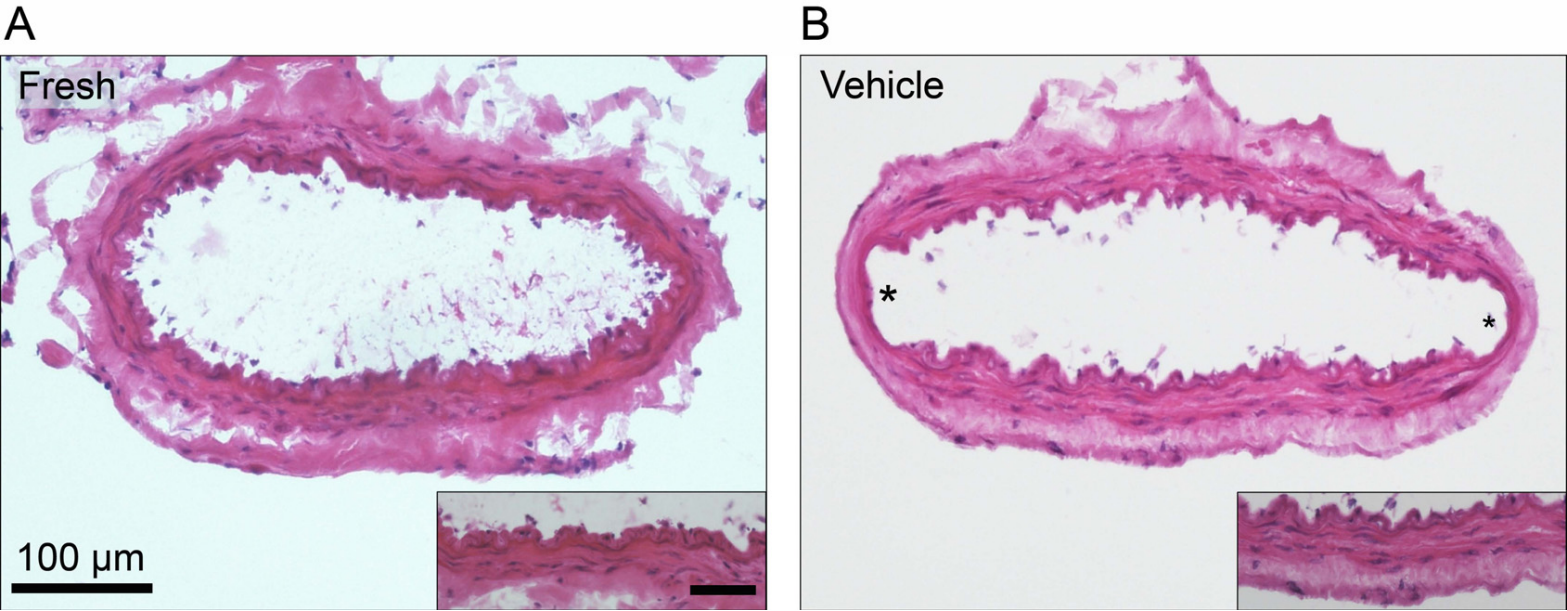
**The effect of organ culture and *in vitro *pharmacology experiments on hematoxylin-eosin staining in human cerebral vessel sections**. (A) Fresh, non-cultured vessel; (B) Vehicle-cultured vessel followed by *in vitro *pharmacology experiment. No morphological changes were observed in the smooth muscle cell layer after the organ culture and *in vitro *pharmacology experiments compared with the fresh vessel sections. Two compressed areas from the wires during the *in vitro *pharmacology experiments were observed (*). These areas were not used for evaluation or analysis. Data were obtained with light microscopy. Insert: higher magnification, scale bar 50 μm.

Hematoxylin-eosin staining was performed on all specimens. No morphological differences were observed in the smooth muscle cell layers except for two compressed areas from the wires in the *in vitro *pharmacology experiments (Figure 2). Therefore, these areas were not used for any kind of evaluation or analysis in the immunohistochemical experiments.

### Expression of G-protein coupled receptors

**Figure 3:**
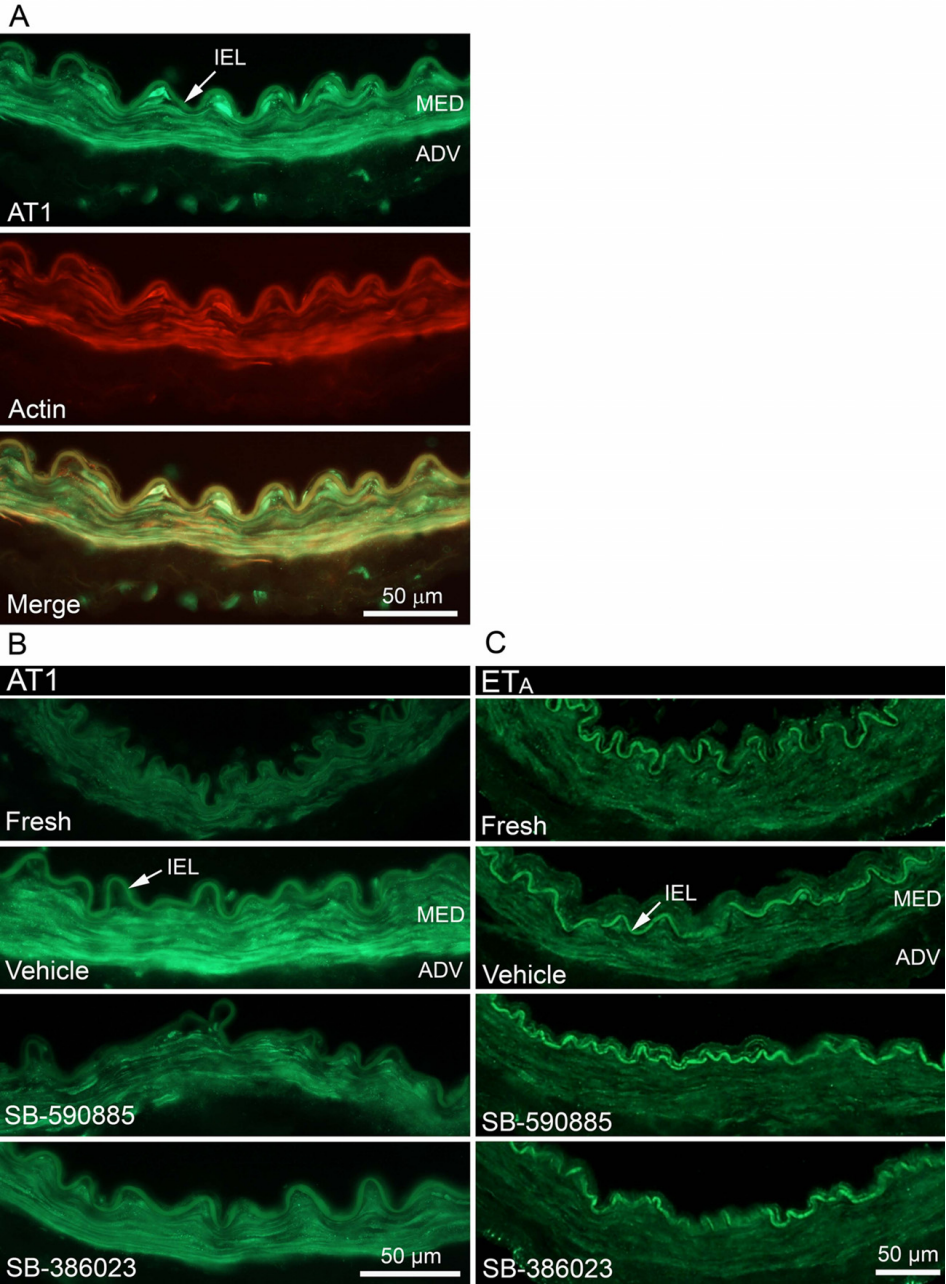
**Human cerebrovascular receptor expression**. (A) Double staining with actin shows expression of angiotensin II type 1 (AT_1_) receptors in the medial layer. (B) AT_1 _receptor expression in fresh and in cultured vessel segments in the presence of vehicle, SB-590885, or SB-386023. The increase in AT_1 _receptor immunoreactivity after culture was diminished significantly after treatment with SB-590885, and diminished slightly after treatment with SB-386023. (C) No difference in endothelin type A (ET_A_) receptor expression was observed. Autofluorescence was observed in internal elastic lamina. Data were obtained with epifluorescence microscopy. MED - medial layer, ADV - adventitial layer, and IEL - internal elastic lamina.

Protein expression of individual receptors was evaluated with immunofluorescence using antibodies against the 5-HT_1B_, AT_1 _(Figure 3A, B), AT_2_, ET_A _(Figure 3C), and ET_B _receptors. In addition, double immunostaining was performed with 5-HT_1B_, AT_1_, and ET_B _receptors together with actin (smooth muscle cell layer) to determine the localization of the receptors. Double staining revealed that all three receptors were found in the smooth muscle cell layer (AT_1_, Figure 3A).

Fluorescence intensity measurements were performed on all receptor stainings. Because of inter-individual differences and differences in pre-treatment and in vessel size, we did not see a close correlation between immunostaining and the *in vitro *experiments. However, a marked increase in AT_1 _receptor immunofluorescence was observed in organ cultured vessels treated with vehicle compared with fresh, non-cultured vessels. The immunofluorescence was reduced in vessels treated with SB-590885, and to a smaller extent after treatment with SB-386023 (Figure 3B), compared with vehicle. No significant differences in 5-HT_1B_, AT_2_, ET_B_, and ET_A _(Figure 3C) receptor immunoreactivity were detected.

### Expression of phosphorylated B-Raf

**Figure 4:**
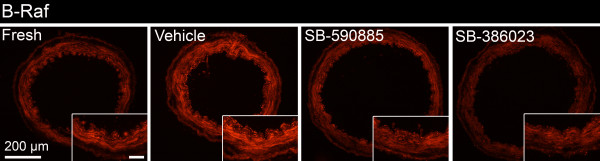
**Phosphorylated B-Raf (p-B-Raf) expression in human cerebral vessels**. Cultured vessel with vehicle only shows increased immunoreactivity compared with fresh, non-cultured vessel. This effect was markedly reduced after treatment with SB-590885 and SB-386023. Data were obtained with epifluorescence microscopy. Insert: higher magnification, scale bar 50 μm.

The protein expression of activated p-B-Raf was evaluated with immunofluorescence. As in the receptor immunofluorescence experiments, inter-individual and segmental differences were observed. Nevertheless, an increase in p-B-Raf immunoreactivity was observed in cultured brain vessels compared with fresh non-cultured vessels. In addition, immunofluorescence intensity was clearly reduced in vessels treated with SB-590885 or SB-386023 (Figure 4).

## Discussion

The present study demonstrates for the first time that upregulation of the contractile receptors 5-HT_1B_, AT_1_, and ET_B _in human cerebral arteries is mediated by B-Raf signaling. These receptor subtypes have been characterized in human cerebral arteries in detail in previous studies [15-17]. In human ischemic stroke, we have observed enhanced levels of protein and mRNA of 5-HT_1B_, AT_1_, and ET_B _receptors in middle cerebral artery smooth muscle cells [5]. Presently, the co-incubation of cerebral arteries with two different B-Raf antagonists prevents organ culture-induced upregulation of contractile responses to 5-CT, Ang II, and ET-1. The effect on receptor-mediated contraction was more prominent after treatment with SB-590885, which is to be expected because it has a lower K_d _value, and therefore a higher affinity for its ligand, than SB-380623 [12,13].

### In vitro pharmacology

The maximum contraction elicited by 5-CT was significantly reduced, as demonstrated by the reduction in E_max _after co-incubation with SB-590885 (Figure 1A, Table 1). The receptor responsible for this contraction has been demonstrated to be the 5-HT_1B _receptor subtype, which is also selectively expressed in human cerebral arteries, as demonstrated by protein 5-HT_1B _expression and inhibition by a selective 5-HT_1B _antagonist [17-19]. The role of 5-HT and its receptors in ischemia are not clear; while some studies report a protective role for 5-HT receptor agonists [20], others show increased contractility and improvement with 5-HT receptor antagonists [21]. Guilbert et al. show that 5-HT_1B _is responsible for the 5-HT aggravation seen in exercise-induced cardiac ischemia in dogs [21]. Additionally, 5-HT_1B _receptors have been suggested to interact with 20-hydroxyeiscosatetraenoic acid (20-HETE) and thereby contribute to the acute fall in regional cerebral blood flow after SAH [22]. We have previously reported on increased 5-HT_1B _protein expression and receptor-mediated contraction after SAH and organ culture in cerebral arteries [14,23], which could impair the cerebral blood flow and thereby contribute to ischemic damage. The present study demonstrates that the selective B-Raf inhibitor SB-590885 significantly decreases 5-HT_1B _receptor-mediated vasoconstriction, suggesting that the elevated contractile response of 5-HT_1B _receptors observed after organ culture is regulated by the B-Raf/MEK/ERK pathway.

Organ culture of isolated human arteries in the presence of SB-590885 or SB-386023 reduced Ang II-mediated contraction (P < 0.001 for SB-590885 treated vessels, Figure 1B, Table 1). Available data show that cerebral vasoconstriction in response to Ang II is mediated by AT_1 _receptors, while vasodilatation is mediated by AT_2 _receptors [24]. The smooth muscle cell AT_1 _receptors are upregulated and show enhanced contractile responses after experimental cerebral ischemia [25] or in human ischemic stroke [5]. In support, the reduced vasoconstrictor responses observed in the present study after treatment with the B-Raf inhibitor SB-590885 lead to a concomitant reduction of the AT_1 _receptor protein when examined immunohistochemically. The receptor identity has been confirmed using selective antagonists for the AT_2 _receptor in human brain vessels [15]. Blockade of the AT_1 _receptor has been shown to improve injury after transient cerebral ischemia [26,27] and to reduce cardiovascular morbidity and mortality in stroke patients [28].

In agreement with a previous study, the selective ET_B _receptor agonist sarafotoxin 6c (S6c) did not elicit any vasoconstrictor responses in cultured human cerebral arteries [16]. Therefore, the high-affinity phase of the ET-1 biphasic concentration-response curve, corresponding to ET_B _receptor-mediated contraction, was studied. The same situation was seen in the rat middle cerebral artery after experimental SAH, detailed pharmacological analysis revealed participation of the ET_B _receptor [29]. In the present study, we demonstrate a significant reduction of the ET_B _maximum contraction after co-incubation with SB-590885; SB-386023 had a weaker effect (Figure 1C, Table 1). No effect on the ET_A _receptor-mediated contraction was observed after treatment with B-Raf inhibitors. It is well known that cerebral vessels have contractile ET_A _receptors in the smooth muscle cells and relaxant ET_B _receptors in the endothelium. However, there is a phenotypic change after stroke in both animals and humans, with the appearance of contractile ET_B _receptors in the smooth muscle cells [5,30]. The effect of selective ET_A _blockers on infarct volume after experimental stroke is ambiguous, with studies showing both effect and no effect [2]. Results have been similar for the combined ET_A _and ET_B _antagonists bosentan and clazosentan. One study using an ET_B _blocker showed an increase in infarct volume [31]. The administration of an ET_B _blocker in conjunction with cerebral ischemia causes a blockade of ET_B _receptor-mediated dilation, which exacerbates the initial vasoconstriction and increases the infarct. The ET_B _blocker might be beneficial if it is administered after upregulation of the ET_B _receptor.

ET receptor antagonists are not the best approach for improving cerebral perfusion after ischemia because of the opposing effects of a strong contractile ET_A _receptor and a dilatory ET_B _receptor. However, a different approach, whereby the signal transduction of the Raf/MEK/ERK pathway was blocked with the MEK1/2 inhibitor U0126, diminished the upregulated ET_B _receptor-mediated contraction and reduced stroke volume [7]. Organ culture of rodent and human cerebral arteries is a way to simulate ET_B _receptor upregulation and to study the molecular mechanisms involved. In the present study, we show that blockade of the MEK/ERK1/2 pathway using upstream B-Raf inhibitors results in attenuated ET_B _receptor-mediated contraction after organ culture.

### Immunohistochemistry

When examined with hematoxylin-eosin staining, no morphological changes were observed in the vessels except for the regions where the steel wires used in the *in vitro *pharmacology experiments have been attached (Figure 2). However, it became obvious in the immunohistochemical examination that the vessels showed considerable inter-individual differences, most likely due to differences between the patients themselves. Some of the patients exhibited more consistent results than others (e.g., repeated immunostainings displayed stable results). These inter-individual differences could explain the inconsistency in the results obtained with the fluorescence intensity measurements.

Immunohistochemical staining using the 5-HT_1B _antibody showed no differences between the groups. In other studies, 5-HT_1B _expression in rat cerebral arteries is increased after middle cerebral artery occlusion and SAH [3,4].

AT_1 _receptor immunoreactivity was reduced after treatment with SB-590885. Previously, increased AT_1 _receptor immunofluorescence after SAH in rats has been shown to be reduced after application of SB-386023 [32]. In our study, we observed a decrease in AT_1 _receptor immunofluorescence intensity after application of SB-590885, but only a small decrease after SB-386023 (Figure 3B), results that are in accordance with the *in vitro *pharmacology experiments.

ET_A _receptor-mediated contractile responses were not significantly altered by the two B-Raf inhibitors used in the present study (Figure 1C). Immunohistochemical examination disclosed the same pattern; no differences were observed between the groups (Figure 3C).

There was an increase in p-B-Raf immunoreactivity after organ culture and this effect could be reduced considerably in the presence of SB-590885 and SB-380623 (Figure 4). Therefore, the activation of B-Raf protein kinase could be blocked by the application of specific antagonists. We suggest that B-Raf is important for the phenotypic changes of GPCRs observed in the smooth muscle cells of cerebral arteries after organ culture and cerebral ischemia [2]. An interesting question is whether B-Raf functions alone or in a heterodimer in this aspect. There is evidence for B-Raf/C-Raf heterodimerization with highly increased kinase activity compared with the respective homodimers or monomers [33]. Further studies are needed to elucidate whether heterodimerization is important for the regulation of GPCRs in vascular smooth muscle cells after ischemia and organ culture.

## Conclusions

In conclusion, we show that selective inhibition of B-Raf using SB-590885 significantly attenuates 5-HT_1B_, AT_1_, and ET_B _receptor-mediated contraction in human cerebral arteries. Therefore, we suggest that B-Raf is important for the altered GPCR expression observed after cerebral ischemia, and that specific blockage might be a novel approach to reduce tissue damage after stroke.

## Methods

### Ethics

This study was approved by the Regional Ethical Review Board in Lund, Sweden (LU-818-01) and has been performed in accordance with the Declaration of Helsinki.

### Tissue collection

Cerebral arteries were obtained from patients undergoing neurological surgery for removal of brain tumor or seizure-producing cortex in severe cases of epilepsy (n = 7, 55 to 77 years old, 4 men and 3 women). Adjacent tissue that was removed contained one or two vessel segments that were used for the experiments. The vessels were dissected during surgery and immediately immersed in cold, sterile Dulbecco's modified Eagle's medium (DMEM, Gibco, Invitrogen, Carlsbad, CA, USA) and transported to the laboratory. The arteries were dissected free from adhering tissue and cut into cylindrical segments approximately 2 mm in length. The outer diameter of the vessels ranged from 250 to 950 μm.

### Organ culture

Arterial segments were cultured for 48 h at 37°C in humidified 5% CO_2 _and air in serum-free DMEM supplemented with penicillin (100 U/ml), streptomycin (100 μg/ml), and amphotericin B (0.25 μg/ml) (Gibco). The method of blood vessel culture and upregulation of contractile receptors has been described previously [34]; the upregulation is not altered with serum present in the medium or by incubation in buffer only, but is a metabolically active process. The segments were cultured in well plates, one segment per well, in the absence or presence of B-Raf inhibitors: SB-386023, 1 μM (K_d _B-Raf: 2.4 nM, GlaxoSmithKline, UK); SB-590885, 1 μM, (K_d _B-Raf: 0.3 nM, GlaxoSmithKline); or the same volume of vehicle (dimethyl sulfoxide). Incubation was also performed with 10 μM SB-590885, which resulted in non-viable arteries when examined by *in vitro *pharmacology; therefore arteries incubated in SB-590855 were not used for further experiments. After 24 h of culture, the DMEM was replaced with new fresh medium and inhibitors or vehicle were added as described above. For each patient and group, one to three vessel segments were incubated and examined by *in vitro *pharmacology and/or immunohistochemistry (see below).

### In vitro pharmacology

For contractile experiments, a myograph was used to record the isometric tension in isolated cerebral arteries [35,36]. The cylindrical segments were threaded on two parallel stainless steel wires (diameter, 40 μm) and mounted in a Mulvany-Halpern myograph (Danish Myo Technology A/S, Aarhus, Denmark). One wire was connected to a force displacement transducer attached to an analogue-digital converter unit (ADInstruments, Chalgrove, UK). The other wire was connected to a micrometer screw, allowing adjustments of the distance between the wires and therefore the vascular tone. Measurements were recorded on a computer by use of a Power Lab unit (ADInstruments). The vessel segments were immersed in temperature-controlled tissue baths (37°C) containing a bicarbonate buffer solution of the following composition (mM): NaCl 119, NaHCO_3 _15, KCl 4.6, MgCl_2 _1.2, NaH_2_PO_4 _1.2, CaCl_2 _1.5, and glucose 5.6. The buffer was continuously aerated with oxygen enriched with 5% CO_2_, resulting in pH 7.4. After an equilibration period of ~20 min, each vessel segment was stretched to 90% of the normal internal circumference, which would be the size each vessel would have if relaxed and under a transmural pressure of 100 mm Hg [36]. The normalization procedure makes certain that all vessel segments are set to a normalized internal circumference giving maximal response. Subsequently, the vessels were allowed to stabilize for 20-30 min. The contractile capacity of the vessels was determined by exposure to an isotonic solution containing 63.5 mM K^+^, obtained by partial change of NaCl for KCl in the above buffer. The contraction induced by K^+ ^was used as reference for contractile capacity [35].

Concentration-response curves were obtained by cumulative application of 5-CT (5-HT_1B _receptor agonist, concentration range 10^-11 ^to 3·10^-5 ^M, Sigma, St. Louis, USA), Ang II (AT_1 _and AT_2 _receptor agonist, concentration range 10^-12 ^to 10^-6 ^M, Sigma) and ET-1 (ET_A _and ET_B _receptor agonist, concentration range 10^-14 ^to 3·10^-7 ^M, Alexis Biochemicals, Farmingdale, NY, USA). The contractile responses to S6c, a selective ET_B _receptor agonist, were tested in a few samples and no contractile responses were observed, in agreement with a previous study [16]. Therefore, the high-affinity phase of the ET-1 concentration-response curve was used to study ET_B _receptor-mediated contraction.

### Immunohistochemistry

Immediately after the *in vitro *pharmacology experiments, the arterial segments were carefully dismantled (n = 4) and embedded in Tissue TEK (Gibco), frozen on dry ice, and kept at -80°C until cryosectioning (10 μm). Additionally, both fresh (non-cultured) and cultured arterial segments (described above) were directly embedded in Tissue TEK without prior *in vitro *pharmacology experiments (n = 3). These arterial segments were used for the ET_A _and ET_B _immunohistochemistry experiments because of the irreversible binding of ET-1 to receptors in the *in vitro *pharmacology experiments that would likely affect antibody-antigen binding [37].

The sections were collected onto Superfrost(r)Plus (Menzel-Gläser, Braunschweig, Germany) glass slides and stored at -80°C until immunohistochemistry. Sections from the *in vitro *pharmacology experiments were stained with hematoxylin-eosin to evaluate vessel morphology and the possible effects of the *in vitro *pharmacology experiments. Thawed sections were fixed for 10 minutes in -20°C acetone and rehydrated in phosphate buffered saline (PBS, pH 7.2) containing 0.25% Triton X-100 (PBST) for 3 × 5 min at room temperature. The sections were then blocked for 1 h at room temperature in blocking solution containing PBS and 5% normal serum (goat or donkey serum, depending on the secondary antibody hosts) to ensure secondary antibody specificity. Subsequently, sections were incubated overnight at 4°C with primary antibodies (Santa Cruz Biotechnology, Santa Cruz, CA, USA): goat anti-human 5-HT_1B _1:100, rabbit anti-human AT_1 _1:100, rabbit anti-human AT_2 _1:100, goat anti-human ET_B _1:150, and rabbit anti-human ET_A _1:50, diluted in PBST containing 1% bovine serum albumin (BSA) and 3% normal serum. Following primary antibody incubation, sections were rinsed in PBS for 2 × 15 min and incubated for 1 h at room temperature with secondary antibodies (FITC-conjugated goat anti-rabbit (Cayman Chemical Co., Ann Arbor, MI, USA) 1:100 or Cy2-conjugated donkey anti-goat (Jackson ImmunoResearch, West Grove, PA, USA) 1:200) in PBST containing 1% BSA and 3% normal serum (goat and donkey serum, respectively). Sections were then rinsed in PBS for 2 × 15 min and mounted in anti-fading mounting medium (Vectashield; Vector Laboratories Inc., Burlingame, CA, USA) or polyvinyl alcohol mounting medium (compatible with Cy2-conjugated secondary antibodies, Sigma). The procedure was repeated on three different occasions to ensure reproducibility. The same experimental procedure was used for negative controls, but primary antibodies were omitted, resulting in no staining in the tissue except for auto-fluorescence in the internal elastic lamina.

Double immunostaining was performed for the 5-HT_1B_, AT_1_, and ET_B _receptors. Antisera against the receptors (described above) were used along with mouse anti-human smooth muscle actin 1:200 (Santa Cruz Biotechnology) and Texas Red-conjugated donkey anti-mouse 1:200 (Jackson ImmunoResearch).

In addition, immunostaining against p-B-Raf was performed on the sections. The protocol mentioned above was used with a primary rabbit anti-human phosphospecific B-Raf antibody 1:25 (Abcam, Cambridge, UK). The secondary antibody used was Cy3-conjugated donkey anti-rabbit 1:400 (Jackson ImmunoResearch).

Immunoreactivity was visualized at the appropriate wavelengths with a light and epifluorescence microscope (Nikon 80i; Tokyo, Japan) and photographed with an attached Nikon DS-2Mv camera. To evaluate colocalization, images from double immunostaining were superimposed in Adobe Photoshop CS (Adobe systems, Mountain View, CA, USA).

### Analysis and statistics

Contractile responses from the *in vitro *pharmacology experiments are expressed as percentage of the contraction induced by 63.5 mM K^+^. The E_max _value represents the maximum contractile response elicited by an agonist and the pEC50 represents the negative logarithm of the drug concentration that elicited half the maximum response. For biphasic responses, E_max __(1) _and pEC_50 (1) _describe the high-affinity phase, and E_max (2) _and pEC_50 (2) _describe the low-affinity phase.

The protein expression of the 5-HT_1B_, AT_1_, AT_2_, ET_B_, and ET_A _receptors in the smooth muscle cell layer of each vessel were analyzed by fluorescence intensity measurements in four areas within each vessel sample (n = 3 to 4 patients in each group) by use of the ImageJ software http://rsb.info.nih.gov/ij. The four areas were chosen after evaluating the hematoxylin/eosin staining results of all specimens.

Data are expressed as mean ± standard error of the mean (s.e.m.), and n refers to the number of patients. Statistical analyses were performed with Kruskal-Wallis non-parametric test followed by Dunn's multiple comparison test, where P < 0.05 was considered significant.

### Study limitations

Human tissue was obtained during neurosurgery for treatment of tumors or epilepsy. Although the surgeons carefully dissected out adjacent non-cancerous or seizure-producing tissue, effects on adjacent tissue cannot be excluded. The collection of human cerebral arteries is performed over a long period of time. Finally, there was great variation in vessel diameter, owing to limited access to human material.

## List of abbreviations

5-CT: 5-carboxamidotryptamine; 5-HT_1B_: 5-hydroxytryptamine type 1 B; Ang II: Angiotensin II; AT_1_: Angiotensin II type 1; ERK1/2: Extracellular signal-regulated kinase 1 and 2; ET-1: Endothelin-1; ET_B_: Endothelin type B; GPCR: G-protein coupled receptor; MEK: Mitogen-activated ERK activating kinase

## Competing interests

The authors declare that they have no competing interests.

## Authors' contributions

HA participated in the design of the study, performed the experiments, analyzed the data, and wrote the manuscript. HS and ON performed the surgeries and reviewed the manuscript. LE conceived the study, guided the experimental procedures, and participated in writing the manuscript.
